# Insomnia – A Heterogenic Disorder Often Comorbid With Psychological and Somatic Disorders and Diseases: A Narrative Review With Focus on Diagnostic and Treatment Challenges

**DOI:** 10.3389/fpsyg.2021.639198

**Published:** 2021-02-11

**Authors:** Bjørn Bjorvatn, Susanna Jernelöv, Ståle Pallesen

**Affiliations:** ^1^Department of Global Public Health and Primary Care, University of Bergen, Bergen, Norway; ^2^Norwegian Competence Center for Sleep Disorders, Haukeland University Hospital, Bergen, Norway; ^3^Department of Clinical Neuroscience, Karolinska Institutet, Stockholm, Sweden; ^4^Department of Psychosocial Science, University of Bergen, Bergen, Norway

**Keywords:** chronic insomnia, cognitive behavioral therapy, sleep apnea, circadian rhythm sleep-wake disorder, restless legs syndrome, parasomnia, hypersomnia, comorbidities

## Abstract

Patients with insomnia complain of problems with sleep onset or sleep maintenance or early morning awakenings, or a combination of these, despite adequate opportunity and circumstances for sleep. In addition, to fulfill the diagnostic criteria for insomnia the complaints need to be associated with negative daytime consequences. For chronic insomnia, the symptoms are required to be present at least 3 days per week for a duration of at least 3 months. Lastly, for insomnia to be defined as a disorder, the sleep complaints and daytime symptoms should not be better explained by another sleep disorder. This criterion represents a diagnostic challenge, since patients suffering from other sleep disorders often complain of insomnia symptoms. For instance, insomnia symptoms are common in e.g., obstructive sleep apnea and circadian rhythm sleep-wake disorders. It may sometimes be difficult to disentangle whether the patient suffers from insomnia disorder or whether the insomnia symptoms are purely due to another sleep disorder. Furthermore, insomnia disorder may be comorbid with other sleep disorders in some patients, e.g., comorbid insomnia and sleep apnea (COMISA). In addition, insomnia disorder is often comorbid with psychological or somatic disorders and diseases. Thus, a thorough assessment is necessary for correct diagnostics. For chronic insomnia disorder, treatment-of-choice is cognitive behavioral therapy, and such treatment is also effective when the insomnia disorder appears comorbid with other diagnoses. Furthermore, studies suggest that insomnia is a heterogenic disorder with many different phenotypes or subtypes. Different insomnia subtypes may respond differently to treatment, but more research on this issue is warranted. Also, the role of comorbidity on treatment outcome is understudied. This review is part of a Research Topic on insomnia launched by Frontiers and focuses on diagnostic and treatment challenges of the disorder. The review aims to stimulate to more research into the bidirectional associations and interactions between insomnia disorder and other sleep, psychological, and somatic disorders/diseases.

## Introduction

This review is part of a Research Topic on insomnia launched by Frontiers and focuses on diagnostic and treatment challenges of the disorder. Short sleep duration and insomnia are associated with a multitude of health problems, such as anxiety, depression, suicidal ideation, obesity, cardiovascular diseases, cancer, dementia, and increased accident risk (Kecklund and Axelsson, 2016; Riemann et al., 2017; Morin et al., 2020). Furthermore, insomnia is prospectively associated with sick leave and work disability (Sivertsen et al., 2009), and in fact, a Norwegian study showed that insomnia complaints contributed to work-related disability as much as, or even more than depression (Overland et al., 2008).

Poor and short sleep can be caused by or associated with a multitude of conditions. In order to guide clinicians in the identification of specific disorders or diseases, the American Academy of Sleep Medicine has developed the International Classification of Sleep Disorders (ICSD), a diagnostic system used within sleep medicine all around the world. In the third and latest version of this classification, ICSD-3 (American Academy of Sleep Medicine, 2014), sleep disorders are classified into six main diagnostic categories: (1) insomnia; (2) sleep related breathing disorders; (3) circadian rhythm sleep-wake disorders; (4) sleep related movement disorders; (5) parasomnias; and (6) central disorders of hypersomnolence (American Academy of Sleep Medicine, 2014; Table 1). Further, each diagnostic category entails several different separate diagnoses, totaling up to more than 50 different sleep disorders. Often, patients may present symptoms characteristic of several sleep disorders when seeking help for their problem, that is, most sleep disorders usually cause disturbances both during the night (e.g., poor and/or interrupted sleep) and during the day (e.g., fatigue, sleepiness, cognitive deficits, mood disturbances). However, even though patients with different sleep disorders may present symptoms or complaints which resemble each other, the treatment-of-choice differs substantially between the different diagnostic categories, as detailed below. Thus, a thorough diagnostic assessment is a necessity in order to be able to best help patients with sleep problems.

**TABLE 1:** Classification of sleep disorders and treatment-of-choice.

Diagnostic category including examples of specific sleep disorders	Treatment-of-choice
Insomnia disorders Chronic insomnia disorder	Cognitive behavioral therapy for insomni

Sleep related breathing disorders Obstructive sleep apnea Central sleep apnea	Continuous positive airway pressure

Circadian rhythm sleep-wake disorders Delayed sleep-wake phase disorder Advanced sleep-wake phase disorder Jet lag disorder	Bright light and/or exogenous melatonin

Sleep related movement disorders Restless legs syndrome Periodic limb movement disorder	Dopamine agonists or alpha2-delta ligands

Parasomnias Sleepwalking (somnambulism) Sleep terrors Nightmare disorder	Sleep hygiene advice

Central disorders of hypersomnolence Narcolepsy Idiopathic hypersomnia	Wake-promoting medications

## Insomnia Disorder

Insomnia is considered the most common sleep disorder (Riemann et al., 2017). Chronic insomnia disorder is defined by subjective complaints with initiating or maintaining sleep, despite adequate opportunity and circumstances for sleep. Furthermore, the complaints need to be present at least 3 days per week for a duration of at least 3 months (American Academy of Sleep Medicine, 2014). In addition, the sleep complaints need to be associated with negative daytime consequences (e.g., fatigue, impairment in concentration or memory, mood disturbance, lack of energy, dissatisfaction with sleep) to fulfill the diagnostic criteria. Short-term (acute) insomnia has similar diagnostic criteria, but the symptoms have a duration of less than 3 months and no requirements are giving concerning number of days per week with presence of symptoms. For both short-term and chronic insomnia disorder, it is mandatory for the diagnosis that the sleep disturbance and associated daytime symptoms are not better explained by another sleep disorder (American Academy of Sleep Medicine, 2014). It is important to note that there are no objective criteria for the diagnosis of insomnia disorder. Thus, polysomnography is not indicated. However, polysomnography may be used to rule out other sleep disorders, e.g., obstructive sleep apnea, and may be used for subtyping insomnia, e.g., insomnia with objective short sleep duration (Vgontzas et al., 2013; American Academy of Sleep Medicine, 2014). The prevalence of both acute and chronic insomnia symptoms in the general population is high and considered to be on the rise (Pallesen et al., 2014; Riemann et al., 2017). Furthermore, insomnia disorder is often a persistent condition (Morin et al., 2020). The prevalence depends on age, sex and socioeconomic status, and several psychological and somatic disorders/diseases occur comorbid with insomnia (Riemann et al., 2017). The strong association between insomnia and depression has received much research interest, and studies suggest that treatment targeting insomnia symptoms will improve outcomes also for symptoms of depression (Blom et al., 2015, 2017). A transdiagnostic approach to treating sleep disturbance across different psychiatric disorders was introduced by Harvey (2009) and such an approach has gained popularity (Norton et al., 2013; Norton and Roberge, 2017; Pearl and Norton, 2017). However, insomnia has traditionally not received much attention in clinical practice. This is highlighted in a study based on three independent and nationally representative data-sets from mental health care centers in Norway, in which the diagnosis of insomnia as a primary or comorbid diagnosis was made only for 34 out of 42,507 patients (0.08%), even though about 40% of the patients experienced severe sleep disturbance and 22% reported this to be one of their most prominent problems (Kallestad et al., 2011).

The treatment-of-choice for chronic insomnia disorder is cognitive behavioral therapy (CBTi), in which the behavioral treatment components (the B in CBTi) sleep restriction and stimulus control are shown to be the most effective components (Morin et al., 1994; Brasure et al., 2016; Riemann et al., 2017). Sleep restriction is a behavioral treatment method in which time in bed is restricted to the actual time the patient sleeps, usually based on estimates from sleep diaries. Patients with chronic insomnia disorder typically spend 8–10 h in bed each night, still often report only an average of 5 or less hours of sleep. Initially, the patient is instructed to stay in bed only for this estimated sleep duration (called the sleep window), e.g., from 02.00 to 07.00 h. Adjustments of the sleep window are made, usually on a weekly basis, depending on the progress. If the patient is asleep during most of the sleep window (e.g., sleep efficiency is above 80–85%), time in bed is increased by 15–30 min, until optimal sleep duration and daytime function are reached. Stimulus control refers to a set of instructions with the aim that the patient should break the often established link between bed/bedroom and negative states (e.g., worry) and again learn to associate bed/bedroom with relaxation and sleep in order to reestablish a proper sleep-wake pattern. The instructions tell the patient not to stay in bed, if sleep does not occur. This means that the patient is instructed to get out of bed when unable to sleep and to stay up until sleepiness resumes. The bedroom should be used only for sleep (and sex), and not for e.g., work, TV watching or use of electronic media. Furthermore, the patient is instructed to set a fixed rise time in the morning. In addition to behavioral treatment components, CBTi also includes cognitive strategies (the C in CBTi) to identify, challenge and change dysfunctional beliefs, attitudes and misconceptions about sleep and insomnia (Riemann et al., 2017). Cognitive factors have been identified as major factors driving insomnia (Harvey, 2002). Many patients with insomnia focus on their complaints and its consequences. Unfortunately, “trying to sleep” is not an effective method and tends to only lead to more insomnia. It has been argued that the attentional system in patients with insomnia may be abnormally sensitive to sleep-related information (Harris et al., 2015). This leads to increased rumination and intention toward sleeping (Harvey, 2002; Espie et al., 2006). Other CBT-strategies such as problem solving, for instance on how to deal with pre-sleep cognitions and cope with stress, can also be included. Other treatment components sometimes included in CBTi comprise sleep hygiene, which is basic advice (e.g., not drink coffee too late) on how to promote sleep, and various relaxation techniques to counteract physiological, emotional and cognitive activation. Unfortunately, CBTi is often not available for patients with chronic insomnia, and most patients still receive treatment in terms of medications and/or general sleep hygiene advice (Cheung et al., 2019). Thus, easily implementable therapies in clinical practice are clearly needed. CBTi delivered through the Internet (Ritterband et al., 2009; Espie et al., 2012) or through self-help books (Bjorvatn et al., 2011; Jernelov et al., 2012) show promising results. Before returning to insomnia, we will present the other main sleep disorder categories briefly.

## Sleep Related Breathing Disorders

The diagnostic group sleep related breathing disorders consist of many different disorders, in which obstructive sleep apnea (OSA) is the most common and well-known (American Academy of Sleep Medicine, 2014). The sleep related breathing disorders are characterized by abnormal respiration during sleep with frequent apneas and/or hypopneas. Patients with OSA typically complain of poor and non-restorative sleep at night and fatigue during daytime. Thus, symptoms may appear similar to insomnia, however, the assessment and recommended treatment differ greatly. Insomnia disorder is a purely subjective diagnosis, based on a clinical interview with the patient, and with no objective criteria. On the other hand, OSA requires objective findings on polysomnographic or polygraphic recordings (American Academy of Sleep Medicine, 2014). In fact, if the apnea-hypopnea index (AHI) is 15 or higher per hour on objective recordings, the diagnostic criteria for OSA are fulfilled even if the patient has no symptoms. If AHI is 5–15 per hour, the diagnostic criteria require some sort of symptoms or signs (e.g., snoring, breathing pauses during sleep, hypertension). The prevalence of OSA is especially high among males, and increases with age (Gabbay and Lavie, 2012) and with increasing weight (Romero-Corral et al., 2010). Treatment-of-choice for moderate to severe OSA is continuous positive airway pressure (CPAP) (Epstein et al., 2009).

## Circadian Rhythm Sleep-Wake Disorders

Circadian rhythm sleep-wake disorders are characterized by a persistent or recurrent pattern of sleep disturbance due to alterations of the circadian timing system (e.g., delayed sleep-wake phase disorder) or a misalignment between the individual endogenous circadian rhythm and exogenous factors influencing the timing, duration and quality of sleep (e.g., jet lag disorder) (Bjorvatn and Pallesen, 2009; American Academy of Sleep Medicine, 2014). Circadian rhythm sleep-wake disorders typically lead to insomnia symptoms and daytime sleepiness and fatigue. Delayed sleep-wake phase disorder is characterized by a significant delay of the major sleep episode evidenced by inability to fall asleep and wake up at a desired or required clock time (American Academy of Sleep Medicine, 2014). However, in contrast to insomnia disorder, patients with delayed sleep-wake phase disorder exhibit normal sleep quality and quantity when allowed to follow their *ad libitum* schedule. Thus, whereas patients with insomnia disorder have problems with sleep independent of time, patients with a circadian rhythm sleep-wake disorder have no problems with sleep *per se*, but they have difficulties with sleeping at the appropriate or desired time of day. In adolescence, delayed sleep-wake phase disorder may be even more prevalent than insomnia disorder (American Academy of Sleep Medicine, 2014). Treatment-of-choice is different from insomnia and consists of scheduled (timed in relation to the patient’s circadian rhythm) administration of bright light and/or exogenous melatonin (Wilhelmsen-Langeland et al., 2013; Saxvig et al., 2014).

## Sleep Related Movement Disorders

Sleep related movement disorders are characterized by repetitive movements that disturb sleep (American Academy of Sleep Medicine, 2014). Of the sleep related movement disorders, restless legs syndrome/Willis-Ekbom disease is the most well-known. This disorder is characterized by an urge to move the legs due to unpleasant sensations. Symptoms begin or worsen during inactivity/rest and are partially or totally relieved by movement. Typically the symptoms present only in the evening or at night (American Academy of Sleep Medicine, 2014). Notably, the diagnosis of restless legs syndrome only requires subjective reports from the patient, hence no objective recordings are required. Patients with severe restless legs syndrome often complain of sleep onset problems, due to the unpleasant sensations, but they also experience poor sleep quality and daytime fatigue/sleepiness. This may result from the fact that restless legs syndrome is closely associated with periodic limb movements during sleep (American Academy of Sleep Medicine, 2014). Thus, symptoms associated with restless legs syndrome may resemble symptoms associated with insomnia disorder. As for insomnia disorder, restless legs syndrome is more common in females compared to males, and the prevalence increases with age (Hening et al., 2007). The treatment-of-choice for severe restless legs syndrome and periodic limb movements during sleep is medications, in which dopamine agonists or alpha2-delta ligands are most commonly used (Liu et al., 2016; Garcia-Borreguero and Cano-Pumarega, 2017).

## Parasomnias

Parasomnias are a group of sleep disorders pertaining to undesirable physical events or experiences that occur during entry into sleep, within sleep or during arousal from sleep (American Academy of Sleep Medicine, 2014). These disorders are divided into those arising from deep sleep [non-rapid-eye movement (NREM) sleep], rapid-eye movement (REM) sleep, and those not arising from any specific sleep stage. Well-known NREM-parasomnias are sleep walking (somnambulism), sleep terrors and sleep-related eating disorder. Nightmare disorder and REM-sleep behavioral disorder are examples of REM-parasomnias (Bjorvatn et al., 2010; American Academy of Sleep Medicine, 2014). Overall, parasomnias often disturb sleep quality and quantity, and lead to daytime fatigue and sleepiness. A majority of the parasomnias are more prevalent in young versus old age, and also in females compared to males and in patients with mental health problems (Bjorvatn et al., 2010; Li et al., 2010). Treatment-of-choice varies depending on severity, but sleep hygiene advice may often be sufficient (Ntafouli et al., 2020).

## Central Disorders of Hypersomnolence

Central disorders of hypersomnolence (hypersomnias) are characterized by excessive daytime sleepiness. Most of these disorders are rare and include severe neurological conditions, such as narcolepsy (Heier et al., 2009; American Academy of Sleep Medicine, 2014). Although the main complaint is excessive daytime sleepiness, patients with narcolepsy may also complain of poor sleep quality and sleep fragmentation. Thus, insomnia symptoms may be present among patients also in this diagnostic category (American Academy of Sleep Medicine, 2014; Thorpy and Bogan, 2020). The assessment and diagnostic criteria of central disorders of hypersomnolence (e.g., narcolepsy) require objective sleep recordings (polysomnography and multiple sleep latency test) or measurement of hypocretin levels in the cerebrospinal fluid (American Academy of Sleep Medicine, 2014). Treatment-of-choice is wake-promoting medications (Thorpy and Bogan, 2020).

## Discussion

Insomnia symptoms may be present in all the different sleep disorders, as highlighted above. Importantly, the diagnostic criteria require that the diagnosis of insomnia disorder should only be used when the symptoms may not be better explained by another sleep disorder. Sometimes it is difficult to disentangle whether the patient suffers from insomnia disorder or whether the symptoms are an indication of another sleep disorder. Furthermore, some patients may suffer from both insomnia disorder and another sleep disorder with insomnia symptoms. For instance, it is not uncommon that patients with OSA also have insomnia disorder, that is, comorbid insomnia and OSA (so-called COMISA) (Sweetman et al., 2019a). Hence, thorough clinical assessment and proper diagnostic evaluation are of great importance, as the final diagnosis reached has serious treatment implications. CBTi is very effective in patients with insomnia disorder but may not be recommended if the insomnia symptoms are purely due to another sleep disorder. In line with this, and due to limited research on the use of CBTi with these patient groups, CBTi and especially the behavioral treatment component sleep restriction, may not be advised in patients with e.g., OSA or delayed sleep-wake phase disorder, as it may cause adverse effects, i.e., increased daytime sleepiness. Also, if the patient suffers from OSA, and it seems likely that the apneas and hypopneas are causing the insomnia symptoms, treatment with CPAP will reduce these symptoms (Bjornsdottir et al., 2013; Mysliwiec et al., 2020). Similarly, if the insomnia symptoms are due to e.g., delayed sleep-wake phase disorder, bright light or exogenous melatonin administration will effectively reduce the insomnia symptoms (Saxvig et al., 2014).

However, as mentioned, sometimes it is difficult to disentangle between insomnia disorder and insomnia symptoms due to another sleep disorder, or whether the patient in fact suffers from comorbidity, that is, both insomnia disorder and another sleep disorder. In line with this, several recent studies show that treatment with CBTi reduces the insomnia complaints in patients with COMISA (comorbid insomnia and OSA) (Sweetman et al., 2017, 2020; Ong et al., 2020) and may also improve CPAP adherence (Sweetman et al., 2019b). Thus, in comorbid conditions, the recommended treatment should be to focus on both sleep disorders (Ong et al., 2020).

To differentiate between insomnia disorder and insomnia symptoms has also important implications when studying prevalence. Several papers indicate that chronic insomnia is the most common sleep disorder (Riemann et al., 2017). However, even though well-validated instruments such as the Insomnia Severity Index (Bastien et al., 2001) and the Bergen Insomnia Scale (Pallesen et al., 2008) are being used, a definite distinction between insomnia disorder and insomnia symptoms due to other sleep disorders (e.g., OSA or a circadian rhythm sleep-wake disorder), is not easy to make in questionnaire-based epidemiological studies. For instance, in a questionnaire study (using the Bergen Insomnia Scale) among patients visiting their general practitioner about 50% of the participants fulfilled the criteria for chronic insomnia (Bjorvatn et al., 2017). However, that study (Bjorvatn et al., 2017) and several other studies (Morin et al., 2006; Hysing et al., 2013; Mallon et al., 2014; Pallesen et al., 2014; Sivertsen et al., 2014) which report prevalences of chronic insomnia are often not able to exclude the possibility that the insomnia symptoms may be better explained by other sleep disorders, such as OSA. Thus, while epidemiological studies indicate a very high prevalence of insomnia disorder, these prevalence numbers do not necessarily indicate how many should be treated with CBTi, as some of these patients with chronic insomnia symptoms may suffer from sleep disorders other than insomnia disorder. We therefore recommend caution when interpreting prevalence of sleep disorders from epidemiological studies, as a definite diagnosis of insomnia disorder should be based on a clinical interview, preferably by a clinician with expertise in sleep medicine. Even in such cases misdiagnosis may occur, and when patients compliant with CBTi do not experience improvement, referral to objective sleep recordings may be warranted. At the same time, under-diagnosing of insomnia, and the use of treatments not following guidelines is a problem, resulting in many patients not receiving adequate treatment for their insomnia (Baglioni et al., 2020).

Traditionally, sleep problems have been regarded as consequences of other underlying conditions such as mental health difficulties or somatic diseases. However, this perspective has changed significantly during the last decade, and recent diagnostic classification systems [e.g., ICSD-3 (American Academy of Sleep Medicine, 2014), DSM-5 (American Psychiatric Association, 2013)] recommend focusing on sleep problems as independent conditions (Riemann et al., 2017). It is now recognized that sleep disorders can no longer be considered only as symptoms, but also as major causative factors of a wide array of problems (Harvey, 2001). In keeping with this, pain has been pointed to as a sleep disturbing factor, but evidence also suggests that poor sleep exacerbates pain (Finan et al., 2013). Sleep disturbance is further recognized as a strong feature of psychopathology as it has been associated with a wide array of psychiatric disorders, such as depression, bipolar disorder, psychotic disorders, anxiety disorders, ADHD, and eating disorders (Sateia, 2009). Although insomnia symptoms are conceivably a part of the diagnostic criteria of several psychiatric disorders (Costantini et al., 2020), it is fairly well demonstrated that insomnia constitutes a risk factor for the development of depression and anxiety in particular (Lovato and Gradisar, 2014; Sivertsen et al., 2014). Furthermore, it is often difficult to conclude whether insomnia symptoms are due to depression or causing depressed mood. Therefore, treatment of both insomnia and depression is recommended when both disorders are present, irrespective of which symptoms came first. Several somatic illnesses, such as diabetes, asthma, epilepsy, rheumatic disorders (Lazaratou et al., 2012), are associated with insomnia. Insomnia may be a result of discomfort caused by the medical condition or it can be caused by the same mechanism as the somatic disorder. On the other hand, insomnia may aggravate somatic disorders, and vicious cycles in which sleep problems and somatic disorders exacerbate each other reciprocally have been described (Lazaratou et al., 2012). Furthermore, sleep can also be impaired as a consequence of treatment regimens related to both psychiatric and somatic disorders. For instance, several antidepressants may disturb sleep (Hauser et al., 2013). For many disorders and conditions where comorbidity with insomnia is present, it has been shown that CBTi can be used to improve sleep (Geiger-Brown et al., 2015), and sometimes also to improve the symptoms of the other condition (Belleville et al., 2011; Blom et al., 2015; Wu et al., 2015).

Several recent studies suggest that insomnia is a heterogenic disorder with different phenotypes or subtypes. For instance, insomnia with objective short sleep duration may be a more severe subtype than other subtypes (Vgontzas et al., 2013). A recent study suggested to divide insomnia into subtypes based on non-sleep characteristics such as life history, mood perceptions, and personality (Blanken et al., 2019). Further, insomnia may be divided into subtypes based on assumed etiology (e.g., psychophysiological, paradoxical, inadequate sleep hygiene), as suggested in the previous version of the International Classification of Sleep Disorders (American Academy of Sleep Medicine, 2005). Insomnia subtypes may also be based on consistency (persistent, remission, relapse) (Wu et al., 2014). In clinical practice, the most commonly used subdivision is related to when in the main sleep period (at sleep onset, during the sleep period, or in the morning) the insomnia symptoms occur (Yokoyama et al., 2010; Bjoroy et al., 2020). In a recent population-based study with more than 60,000 participants, it was shown that the symptoms of anxiety and depression, and hypnotic use, were higher among participants with insomnia consisting of a combination of sleep onset, sleep maintenance, and early morning awakening problems as compared to participants with other insomnia subtypes (e.g., insomnia with pure sleep onset or sleep maintenance problems) (Bjoroy et al., 2020).

In clinical practice, different subtypes may respond differently to treatment (Riemann et al., 2017). For instance, CBTi may be more effective in patients with sleep onset problems than among patients with pure early morning awakening; hence more research on different insomnia subtypes is warranted. The role of comorbid conditions, both psychological and somatic, on treatment outcomes is also understudied. One goal of this review is therefore to inspire research into the bidirectional associations and interactions between insomnia and other psychological and somatic conditions.

An important point to make is that subtyping insomnia symptoms may be helpful in the diagnostic assessment. For instance, sleep onset problems *per se* are not typical in patients with OSA. Patients with OSA more commonly complain of nocturnal awakenings and non-restorative sleep (Bjornsdottir et al., 2013). Thus, if a patient with OSA also has severe sleep onset problems, the presence of a comorbid insomnia disorder is more likely than if the patient mainly complains of sleep maintenance problems. Similarly, if a patient has severe sleep onset problems but no problems maintaining sleep, and is able to sleep for more than 7–8 h, a delayed sleep-wake phase disorder is more likely than an insomnia disorder (Bjorvatn and Pallesen, 2009).

Although CBTi may not, based on current (lack of) evidence, be indicated in cases where insomnia symptoms clearly are secondary to another condition, clinicians should still be aware that insomnia symptoms may prevail (e.g., due to irrational compensatory behavior and negative conditioning) after successful treatment of the main disorder. In cases where the clinician prioritizes treatment of the main disorder expecting this also to provide an indirect effect on sleep, he or she should keep track of how the patient is sleeping. If sleep problems persist following successful treatment of the main disorder, treatment focus should then be on the insomnia symptoms.

In conclusion, patients often present similar symptoms irrespective of sleep disorder, that is, they complain of poor sleep and impaired daytime function. However, as treatment-of-choice varies substantially between the different sleep disorders, it is of great importance that clinicians are able to separate insomnia disorder from other sleep disorders. A thorough diagnostic assessment is thus crucial. At the same time, co-morbidity with other disorders and conditions is high, insomnia is often not recognized in healthcare, and CBTi, which is the recommended treatment, is often not provided. Insomnia symptoms may represent a separate disorder, be symptoms of another sleep disorder, or represent a comorbid disorder. We recommend treatment dependent on the diagnosis being made, that is, if insomnia disorder is present, either as the only diagnosis or as a comorbid disorder, treatment with CBTi is recommended.

## Author Contributions

BB drafting the manuscript. SP and SJ critical review of the manuscript. All authors have read and approved the final version of the manuscript.

## Conflict of Interest

The authors declare that the research was conducted in the absence of any commercial or financial relationships that could be construed as a potential conflict of interest.
